# The renin–angiotensin–aldosterone-system in sepsis and its clinical modulation with exogenous angiotensin II

**DOI:** 10.1186/s13054-024-05123-7

**Published:** 2024-11-26

**Authors:** Matthieu Legrand, Ashish K. Khanna, Marlies Ostermann, Yuki Kotani, Ricard Ferrer, Massimo Girardis, Marc Leone, Gennaro DePascale, Peter Pickkers, Pierre Tissieres, Filippo Annoni, Katarzyna Kotfis, Giovanni Landoni, Alexander Zarbock, Patrick M. Wieruszewski, Daniel De Backer, Jean-Louis Vincent, Rinaldo Bellomo

**Affiliations:** 1 Department of Anesthesia and Perioperative Care, Division of Critical Care Medicine, University of California San Francisco, 521 Parnassus Avenue, San Francisco, CA 94143 USA; 2 Department of Anesthesiology, Section on Critical Care Medicine, Wake Forest School of Medicine, Atrium Health Wake Forest Baptist Medical Center, Winston-Salem, NC USA; 3 Department of Critical Care, Guy’s and St Thomas’ Hospital, London, UK; 4 Department of Intensive Care Medicine, Kameda Medical Center, Kamogawa, Japan; 5 Department of Intensive Care, Department of Medicine, SODIR Research Group, VHIR, Vall d’Hebron University Hospital, Universitat Autònoma de Barcelona, Barcelona, Spain; 6 Anesthesia and Intensive Care Department, University Hospital of Modena, University of Modena and Reggio Emilia, Modena, Italy; 7 Department of Anesthesiology and Intensive Care Unit, Nord Hospital, Aix Marseille University, Assistance Publique Hôpitaux Universitaires de Marseille, Marseille, France; 8 Dipartimento di Scienze Biotecnologiche di Base, Cliniche Intensivologiche e Perioperatorie, Università Cattolica del Sacro Cuore, Rome, Italy; 9 Dipartimento di Scienze dell’Emergenza, Anestesiologiche e della Rianimazione, Fondazione Policlinico Universitario A, Gemelli IRCCS, Rome, Italy; 10 Department of Intensive Care Medicine, Radboud UMC Nijmegen, Nijmegen, The Netherlands; 11 Pediatric Intensive Care and Neonatal Medicine, Bicêtre Hospital, AP-HP Paris Saclay University, Le Kremlin-Bicêtre, Paris, France; 12 Department of Intensive Care, Erasme University Hospital, Université Libre de Buxelles, Brussels, Belgium; 13 Department of Anaesthesiology, Intensive Therapy and Pain Medicine, Pomeranian Medical University, Szczecin, Poland; 14 Department of Anesthesia and Intensive Care, IRCCS San Raffaele Scientific Institute, Milan, Italy; 15 School of Medicine, Vita-Salute San Raffaele University, Milan, Italy; 16 Department of Anesthesiology, Intensive Care and Pain Medicine, University Hospital of Münster, Albert-Schweitzer Campus 1, Building A1, 48149 Münster, Germany; 17 Department of Pharmacy, Mayo Clinic, Rochester, MN USA; 18 Department of Anesthesiology, Mayo Clinic, Rochester, MN USA; 19 Department of Intensive Care, CHIREC Hospitals, Université Libre de Bruxelles, Brussels, Belgium; 20 Department of Intensive Care, Austin Hospital, Melbourne, Australia; 21 Department of Critical Care, The University of Melbourne, Melbourne, Australia; 22 Australian and New Zealand Intensive Care Research Centre, Monash University, Melbourne, Australia

## Abstract

Dysregulation of the renin–angiotensin–aldosterone-system (RAAS) in sepsis is a complex and early phenomenon with a likely significant contribution to organ failure and patient outcomes. A better understanding of the pathophysiology and intricacies of the RAAS in septic shock has led to the use of exogenous angiotensin II as a new therapeutic agent. In this review, we report a multinational and multi-disciplinary expert panel discussion on the role and implications of RAAS modulation in sepsis and the use of exogenous angiotensin II. The panel proposed guidance regarding patient selection and treatment options with exogenous angiotensin II which should trigger further research.

## Introduction

The renin–angiotensin–aldosterone-system (RAAS) is, together with the sympathetic nervous system and vasopressinergic system, one of the three dominant blood pressure-regulating mechanisms in the human body [1]. The RAAS has been a therapeutic target in the treatment of hypertension for decades and has been extensively investigated in such settings to find strategies blocking its effect on blood pressure. In contrast, its blood pressure maintaining role in vasoplegic states in general and in septic vasodilatory shock in particular has only recently regained attention. While targeting mean arterial blood pressure above 65 mmHg has not led to improved outcomes in septic shock [2, 3], vasopressors remain a key component of the treatment of septic shock, and there is no doubt that profound hypotension should not be left untreated.

The regulatory approval of angiotensin II as a vasopressor agent for treating vasodilatory shock offers a therapeutic option to modulate the RAAS in septic shock. For most clinicians familiar with the inhibition of RAAS, augmenting its effect by administering intravenous angiotensin II might appear counterintuitive. This requires appropriate explanation and education.

Moreover, since the ATHOS-3 trial [4], there has been a marked increase in the understanding of the pathophysiology and intricacies of the RAAS in septic vasodilatory shock and its response to therapy, the role of renin, the selection of target populations and early identification of likely responders. Accordingly, we provide the summary of a multi-disciplinary expert panel report with a state-of-the-art update for clinicians regarding the RAAS in septic shock. We discuss the potential use of exogenous angiotensin II as a vasopressor and provide guidance regarding patient selection and treatment modalities. Finally, we provide an insight into preliminary new data with alterations in conventional and alternative pathways in the enzyme-peptide cascade that may allow for future areas of research and intervention.

## The pathophysiology of the RAAS in septic shock

**Fig. 1:**
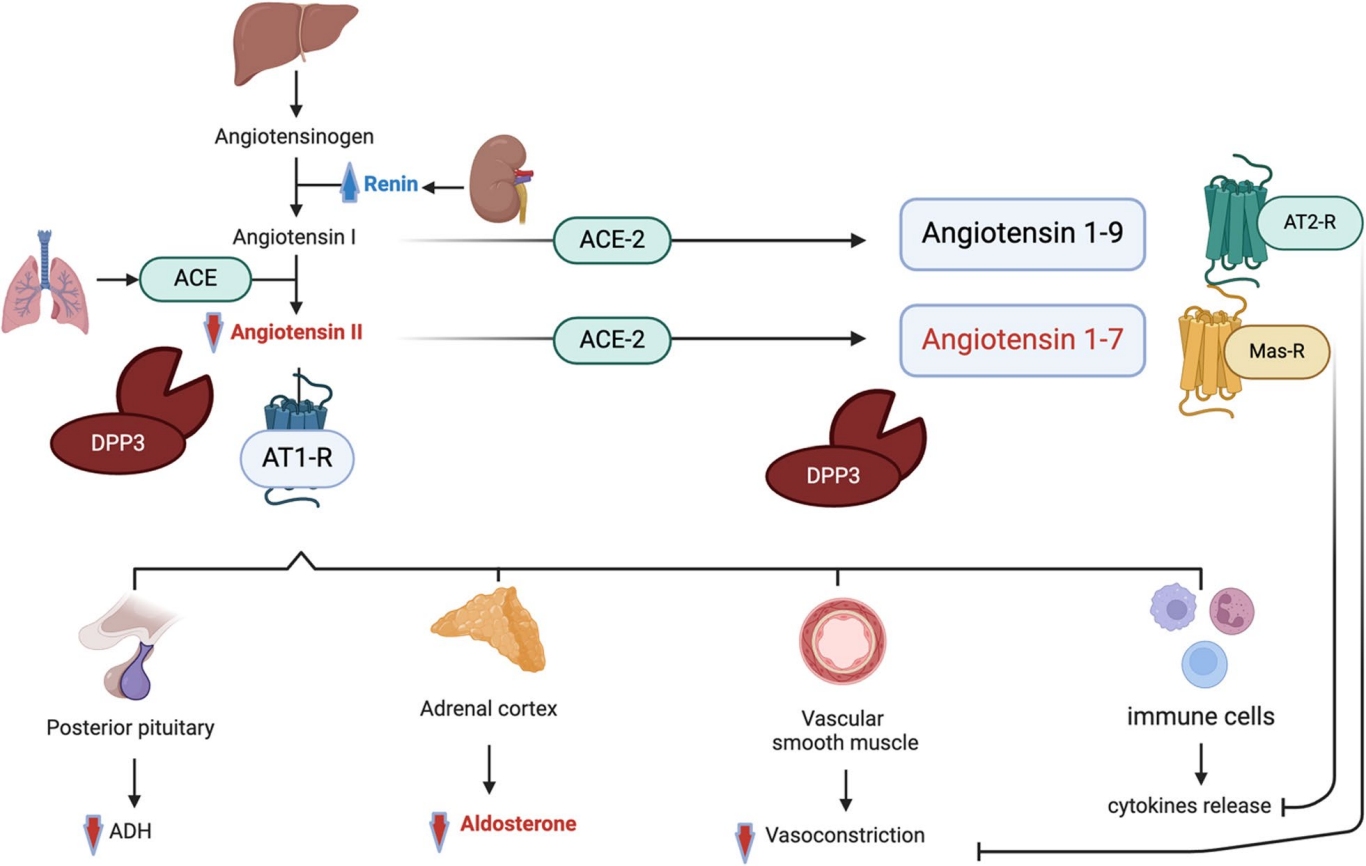
An evidence-based explanatory diagram illustrates our current understanding of RAAS in sepsis. ACE, Angiotensin-converting enzyme; AT1-R, Angiotensin receptor-1; AT2-R, Angiotensin receptor-2; DPP3, Dipeptidyl peptidase 3; ADH, Antidiuretic hormone. DPP3 contributes to regulation of the RAAS by degrading circulating angiotensin peptides (e.g. angiotensin II and angiotensin 1–7)

**Fig. 2:**
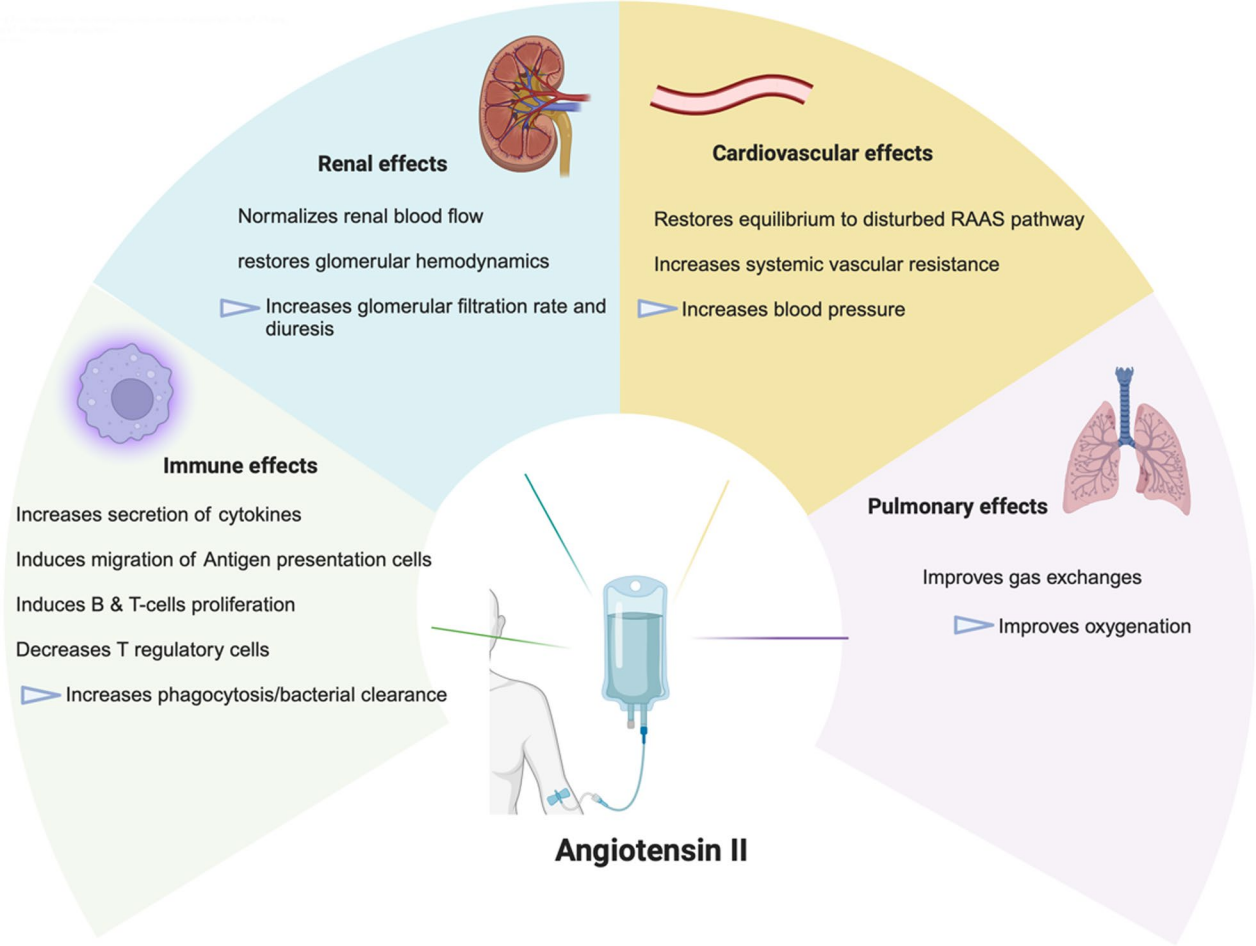
Summary of effects of angiotensin II on organ systems in septic shock

The RAAS has an important role among the physiological systems affected by distributive shock. The dysregulation of the RAAS in sepsis is a complex and early phenomenon with a likely significant contribution to organ failure and patient outcomes (Figs. 1, 2) [1].

### Deficit of angiotensin II type 1 receptors (AT1R) in sepsis

Experimental animal studies utilizing various sepsis models have provided further insight into the pathophysiology of sepsis-related acute kidney injury (AKI) and the role of the RAAS. In a hyperdynamic sepsis model in ewes induced by the intravenous administration of live *E. coli*, hyperdynamic sepsis was associated with increased renal blood flow (RBF) and a fall in glomerular filtration rate (GFR). In this model, Angiotensin II (Ang II) infusion restored systemic arterial pressure and reduced RBF back to baseline, suggesting that the remaining AT1R were not saturated at baseline—likely due to the decreased expression of Angiotensin II (i.e. relative deficiency). Vasodilation appears to be partially driven by a relative deficit of angiotensin type 1 receptors (AT1Rs) in renal tissue in sepsis. This finding was also observed in post-mortem kidneys from patients who died from septic shock [5].

### Effects of AT1R activation on inflammation and immune function

Activation of AT1R also modulates inflammation and innate immune function in sepsis and septic shock. Activation of myeloid lineage cells via AT1R leads to the production of reactive oxygen species, activation of NF-κB, and the production of pro-inflammatory cytokines and interferon (IFN). Following lipopolysaccharide (LPS) stimulation, the production of tumor necrosis factor (TNF) and macrophage inhibitor protein (MIP) is augmented at 24 h, while at 48 h, the productions of TNF, interleukin (IL)-6, IL-1, IL-10, and MIP are down-regulated, suggesting a dynamic role for the AT1R in modulating the immune response during sepsis [6]. While some animal models suggested that blocking the AT1R using angiotensin receptor blockers (ARBs) could reduce inflammation and improve survival [7, 8], it appears unlikely that short-term RAAS activation in humans leads to organ damage and that RASS inhibition during critical illness improves outcomes. Of note, a randomized trial showed more harm associated with ARBs in patients with COVID compared to placebo [9]. In non-inflammatory conditions, studies indicate that AT1R activation by Ang II facilitates the release of norepinephrine, while the decrease in AT1R expression is accompanied by reduced vasopressin production [10, 11].

### Dysregulation of the RAAS in acute kidney injury

Multiple studies have shown that critically ill patients with increased plasma renin concentration are more likely to develop organ failure, including acute kidney injury (AKI) or the composite outcome of major adverse kidney events (MAKE), and are more likely to die. In addition, a baseline renin circulating level and its change is more predictive of poor outcomes compared with lactate [12]. These associations are particularly notable in patients experiencing distributive or septic shock, both in adult [13–16] and pediatric populations [17, 18]. Also, low plasma aldosterone concentration and below normal aldosterone to plasma renin activity ratio have been associated with a higher incidence of AKI, need for renal replacement therapy (RRT), and prolonged length of stay in the intensive care unit (ICU) [19]. In distributive shock, an elevation in Angiotensin I (Ang I) levels and an increased Ang I/II ratio (implying a deficit in the conversion of Ang I to Ang II) compared to healthy volunteers have also been reported [20]. High Ang I/II ratios have been associated with higher vasopressor requirements, increased incidence of development of AKI, worse oxygenation, and increased mortality in distributive shock settings [14, 20, 21]. Together with preclinical data [22–25], this suggests that RAAS dysregulation is a key pathophysiologic component of vasodilatory shock, which is associated with organ failure and impaired outcomes in sepsis. Post-hoc analyses of the ATHOS-3 data have demonstrated that high renin concentrations are expected in non-cardiogenic, non-hypovolemic, vasodilatory shock (i.e., patients with cardiac index of greater than 2.3 L/min/m^2^ or as central venous oxygen saturation > 70% coupled with central venous pressure of more than 8 mm Hg) [14, 20]. This is probably partially secondary to decreased Ang II feedback inhibition in this setting since exogenous Ang II infusion has been seen to decrease renin levels [14]. While angiotensin II is the critical peptide involved in glomerular hemodynamics and filtration regulation, there are no head-to-head studies with vasopressin investigating the impact on renal function.

### RAAS dysregulation in acute lung injury and acute respiratory distress syndrome (ARDS)

The role of RAAS in acute lung injury and the development of ARDS is controversial. Evidence shows that angiotensin-converting enzyme (ACE) activity is altered in pneumonia and ARDS. Experimental inflammatory lung injury causes pulmonary endothelial ACE-1 shedding [26, 27], and pulmonary AT1R signaling may promote ventilator-induced acute lung injury [28–30].

Previous studies have explored the vasoactive effects of catecholamines and Ang II on the lung vasculature [31, 32]. Ang II acts as a selective pulmonary artery vasoconstrictor. Under normoxic conditions, the lung circulation appears more sensitive to the vasoconstrictive effect of Ang II than the systemic circulation [33, 34].

### Interactions of the conventional and the alternative RAAS system

Septic shock states are associated with dysfunction in the conventional RAAS pathways. Hypotension, hypoperfusion, and lack of endogenous Ang II are the usual stimuli for renin production from the juxtaglomerular apparatus of the kidney. A counterregulatory system, termed the alternative RAAS, includes angiotensin-converting enzyme 2 (ACE2) and angiotensin-(1–7) [Ang-(1–7)], or peptidases and contributes to modulating the RAAS [35] (Fig. 1). Patients with septic shock typically have a combination of increased renin and decreased ACE, along with a reduced conversion of Ang I to Ang II, thereby setting up a state of relative Ang II deficit [13, 26, 36]. Recent data has also highlighted the role of the alternative or counterregulatory RAAS in critical illness and provides essential insights and opportunities for potential therapeutic interventions [35]. Upregulation of tissue ACE2 expression and Mas receptor (MasR) was reported in response to LPS-induced AKI [37, 38]. Similarly, altered expression of urine ACE2 was reported in patients with AKI compared to those without AKI [39, 40]. In acute respiratory failure related to coronavirus disease (COVID-19), upregulation of ACE2 in the lungs was reported, accompanied by increased concentrations of Ang-(1–7), a key player of the alternative RAAS with immunomodulation properties [41, 42]. Notably, a recent post-hoc analysis of a sepsis and septic shock population reported increased ACE2 activity, along with decreased ACE and Ang-II, however did not show an association of Ang-(1–7), ACE2 activity, Ang-II, and ACE with mortality [43]. In an experimental model of sepsis, the administration of Ang-(1–7) attenuated kidney injury [44]. The presence of an endogenous ACE inhibitor in septic shock has also been suggested, with no detectable ACE activity despite an increase in the circulating levels of the ACE protein in a critically ill pediatric population [17]. Finally, elevated concentrations of circulating dipeptidyl peptidase 3 (DPP3), a cytosolic metalloprotease that is released into the bloodstream following cell death, are involved in the regulation of the alternative and classical RAAS and associated with mortality and MAKE following sepsis or burn injury [45]. DPP3 is primarily responsible for the hydrolysis of Ang-(1–7) into Ang-(3–7) and a rapid conversion to Ang-(5–7). In addition, this metalloprotease also acts on Ang II to cleave it into Ang IV (Ang-(3–8)), and then Ang-(5–8). Ang IV is also vasodilatory in action and has other cardioprotective and natriuretic effects via the angiotensin II Type IV receptor (AT4R) [46]. Modulating the alternative RAAS may have a potential role in the future. Appropriately controlled studies with precise sampling techniques are needed that target these mechanisms.

## Angiotensin II and its role in clinical modulation of different phenotypes of septic shock

In the ATHOS 3 trial, 344 patients with vasodilatory shock (the vast majority being septic in etiology) were randomized to Ang II or placebo with continuation of background vasopressors. There was a significant increase in blood pressure. In about 70% of patients randomized to receive Ang II, the mean arterial pressure (MAP) rose to 75 mmHg or 10 mmHg above baseline, compared to only 23% in the placebo arm. In addition, background catecholamine usage was significantly decreased in patients receiving Ang II [4]. Beyond the initial phase of treatment initiation, the MAP was not different between groups. However, the group treated with angiotensin II had lower requirements for other background vasopressors (especially norepinephrine) compared to the placebo group.

### Septic shock and AKI, with and without renal replacement therapy (RRT)

Post-hoc data analysis from the ATHOS 3 trial suggests that patients with vasodilatory shock receiving RRT at the time of randomization had improved survival and a shorter duration of RRT when treated with Ang II, compared to norepinephrine alone [47]. The small number of patients and the confounder should, however, make this observation exploratory.

### Septic shock and ARDS

Angiotensin II administration was associated with improved oxygenation versus placebo among patients with ARDS and vasodilatory shock, possibly by improving ventilation-perfusion (V/Q) matching [21, 48]. The potential underlying mechanisms include catecholamine-sparing effects and improved V/Q matching. These findings confirm previous observations of a positive impact of Ang II on blood pressure and gas exchanges in critically ill patients with COVID-19 [49, 50]. A retrospective multicenter study of patients requiring mechanical circulatory support for cardiac failure also showed that Ang II appeared to increase mean arterial pressure (MAP) and decrease other vasopressor requirements without negatively impacting cardiac function or pulmonary pressures [51]. Of note, we recommend that clinicians remain cautious with the use of Angiotensin II in patients with low cardiac output or signs of inappropriate oxygen delivery (i.e., low central venous oxygen saturation). In line with the inclusion criteria of ATHOS-3, patients with a low cardiac output despite fluid resuscitation are likely to benefit from inotropic agents before starting angiotensin-II.

### Septic shock in patients treated with ACEi/ARB/RAAS modulating drugs

A post-hoc sub-group analysis of the ATHOS 3 trial showed that chronic ACE inhibitors and angiotensin receptor blockers (ARBs) differentially alter the response to Ang II treatment in vasodilatory shock [52]. Prior ACE inhibitor exposure, leading to ACE dysfunction and less Ang II production, was associated with increased Ang II responsiveness. In contrast, ARB exposure, leading to Ang II receptor blockade, was associated with decreased responsiveness. Among patients receiving Ang II, the ARAMIS 1 study showed that recent RAAS inhibitor exposure was associated with higher baseline renin levels [53]. Such higher renin levels were further associated with decreased Ang II responsiveness if ARBs were used. Higher renin levels at 24 h despite Ang II infusion in these patients on ARBs were associated with fewer days alive and RRT-free days.

### Septic shock and liver dysfunction

Patients with liver failure with a Model for End-Stage Liver Disease (MELD) score of ≥ 30 were excluded from the ATHOS 3 trial. Still, there was no difference in liver-related adverse events between the Ang II and placebo groups [4]. Given its pharmacokinetic (PK) and pharmacodynamic (PD) characteristics, liver dysfunction is unlikely relevant to the safety and efficacy of Ang II. The safety and effectiveness of Ang II treatment in liver transplant patients are being investigated [54].

### Post-cardiac surgery vasoplegia

A systematic review of 15 studies comprising 195 patients with shock, of whom 90% had post-cardiac surgery vasoplegia and some had septic shock, showed that 156 patients (80%) had received treatment with Ang II [55]. Ang II administration was associated with a higher MAP, decreased vasopressor requirements, and minimal reported adverse events.

### Septic shock in patients with previous thromboembolic events

In the ATHOS 3 study [4], 3/163 patients in the Ang II arm versus 0/158 patients in the placebo group were diagnosed with deep vein thrombosis (DVT). Pulmonary embolism (PE) was not reported as an adverse event. Thrombocytopenia occurred in 16/163 Ang II-treated and 11/158 placebo-treated patients. Ischemic stroke was reported in 1/163 Ang II-treated versus 0/158 placebo-treated patients, acute myocardial infarction in 2 (Ang II) vs. 3 (placebo) patients, and intestinal ischemia was observed in 1 (Ang II) vs. 3 (placebo) patients. Thus, these numbers are too small to come to meaningful conclusions. Given the three vs. 0 patients with a DVT, the use of Ang II in patients with a previous thromboembolic event might theoretically induce an additional risk. In these patients, the risks of hemodynamic instability and high norepinephrine infusion rates must be weighed against the potential risk of a thromboembolic event. Patients with a recent thromboembolic event are likely to receive (more intensive) thromboprophylaxis treatment. The risk of a thromboembolic event during Ang II treatment under these conditions is unknown.

#### Proposed guidance for the use of Angiotensin II

**Fig. 3:**
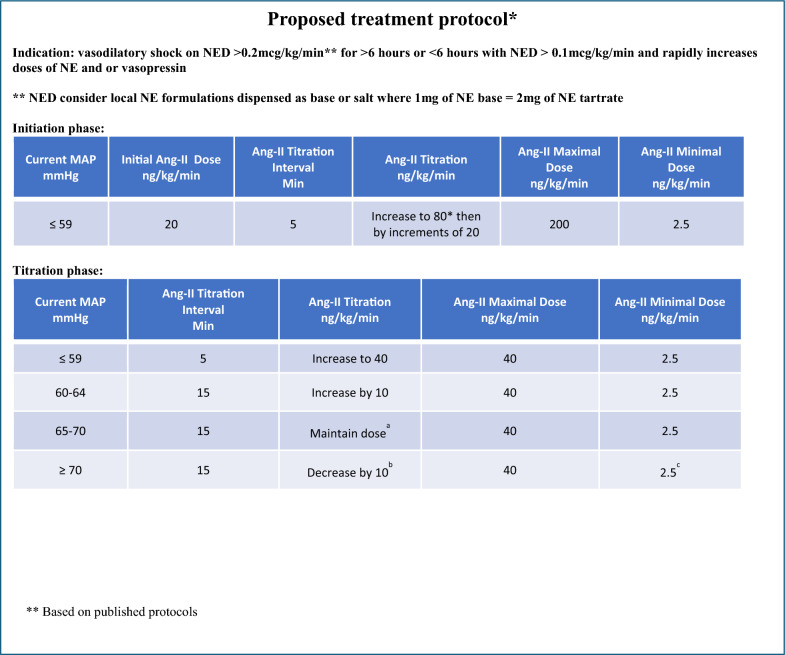
Proposed treatment protocol, *norepinephrine dose is expressed in norepinephrine base

The expert panel agrees that recently published data suggests early initiation of secondary and tertiary vasopressors is beneficial. Based on the ATHOS 3 trial, we suggest considering initiation of Ang II in patients receiving vasopressors at a norepinephrine equivalent dose (NED) of > 0.2 mcg/kg/min and without reduced cardiac output, for at least 6 h [56]. Initiation at a lower dose of background NED for a shorter duration may be considered in shock with rapidly escalating doses of norepinephrine, vasopressin, and other vasoactive agents. We suggest following the ATHOS 3 titration protocol for specific initiation (first 3 h) and titration) (Fig. 3) with a simplification that the usual starting dose of Ang II be 20 ng/kg/min with small titrations of 5 ng/kg/min every 5 min to keep MAP at 65 mmHg or greater. In general, we suggest that background NED response be used to guide therapy. If NED can be lowered considerably, we suggest staying at Ang II of 20 ng/kg/min but increasing by 10 ng/kg/min (the current package insert suggests a maximum of 80 ng/kg/min for a maximum of 3 h and a maximum of 40 ng/kg/min after that) if NED requirement is increasing. It is important to note that in ATHOS 3, the maximum rate of administration was equivalent to a dose of 200 ng/kg/min during the first 3 h and 40 ng/kg/min during the following hours. However, our dosing suggestions are based on the Food and Drug Administration (FDA) drug package insert for the agent in the United States. If blood pressure remains low despite a maximum dose of Angiotensin II, we recommend ruling out and correcting newly developed cardiac dysfunction or hypovolemia and increasing other background vasopressors in case of persistent hypotension due to vasoplegia. An “angiotensin II” challenge test will rapidly differentiate responders from non-responders. Of note, while non-responders are more likely to have a worse outcome than non-responders, Angiotensin II has not been found to worsen outcomes compared to placebo, including in non-responders. Of note, adverse events were numerically less in the angiotensin II group compared to the placebo group in the ATHOS-3 trial (87.1% vs. 91.5%). Moreover, adverse events leading to study drug discontinuation also occurred less frequently in the angiotensin II group (14.1% vs. 21.5%). Regarding risks, adverse events, and serious adverse events, specifically, the risk of thromboembolism with angiotensin II has been a topic that has been extensively debated. A recent systematic review that has examined observational and randomized trial data for angiotensin II after ATHOS-3 has not been able to link or refute a risk of thromboembolism with this agent [57].

Bedside assessment with capillary refill time, skin perfusion, lactate, urinary output, hemodynamics, cardiac output, and other markers as clinically indicated should be considered for tailored hemodynamic management. Ang II is likely to be best used according to clinical need; however, the total duration of infusion should not exceed 7 days and is expected to be around 48 h for most patients. No clinical data informs a specific tapering schedule, which vasopressor to decrease first, and how this relates to a clinical benefit. Nevertheless, clinical guidance is needed as this is relevant for every patient treated with Ang II. For example, the expert panel suggests that in patients on norepinephrine and Ang II who are hemodynamically improving, norepinephrine should be decreased first to an infusion rate considered safe or acceptable (such as 0.1 mcg/kg/min). Subsequently, the Ang II infusion rate should be reduced in steps of 5 ng/kg/min as long as the norepinephrine infusion rate stays the same safe or acceptable rate. If, at any stage, the norepinephrine infusion rate needs to go up again (e.g., > 0.1 mcg/kg/min), Ang II should not be lowered further, or the previous (higher) infusion rate should be restarted.

## Future directions

Currently, responsiveness to Ang II may be best judged clinically by a blood pressure increase. However, future guidance of Ang II therapy in individual patients may necessitate accurate and point-of-care biomarker assays to enrich populations likely to benefit and discern those who may not get any therapeutic benefit or may be harmed. A comprehensive and serial assessment of the RAAS cascade could include the alternative or non-traditional ACE2, DPP3 pathway and the ratios of these changes to understand the most sensitive and specific marker of RAAS dysfunction to best guide treatment. Potential biomarkers include active renin, ACE, ACE-2, DPP3, Ang-(1–7), Ang II concentration, and the Ang-(1–7)/ Ang II ratio. As previously discussed, a post-hoc analysis of the Vitamin C, Thiamine, and Steroids in Sepsis (VICTAS) trial showed that patients with high baseline (compared to population median) or increasing renin levels had a worse overall survival probability compared to those with low baseline or decreasing renin levels (i.e., prognostic enrichment) [43]. RAAS measures and changes in RAAS components following initiation of Ang II treatment may also predict therapeutic efficacy on clinical endpoints [20]. Serum renin concentration is markedly elevated in catecholamine-resistant vasodilatory shock. It may identify patients for whom treatment with Ang II benefits clinical outcomes (i.e., predictive enrichment) [14]. Additionally, several clinical studies have shown that Ang II-dependent hyperactivation of the RAAS axis represents a significant contributor to endothelial dysfunction and organ damage in chronic cardiovascular conditions [58]. In septic shock, different phenotypes have been identified using latent class analysis, with a hyperinflammatory phenotype characterized by higher levels of proinflammatory cytokines, markers of organ dysfunction, and endothelial injury [59]. Future studies should investigate the impact of Ang II infusion on the hyperactivated inflammatory cascade, possibly addressing heterogeneity of treatment effect across different septic phenotypes. The impact of Ang II on fluid resuscitation, organ perfusion and the microcirculation should be explored in patients [60]. Finally, future studies should investigate the impact of Ang II on patient-centered outcomes.

An obvious next step would be a trial with a high renin septic shock population (predictive enrichment) where the effect of Ang II will be compared with standard-of-care vasopressors and followed with timed biomarker levels. Exploring the impact of the use of Ang II at lower doses of first-line vasopressors and the timing of initiation is also warranted. A prespecified post-hoc analysis of the ATHOS 3 trial showed some patients are exquisitely responsive to very low doses of Ang II, and that down-titration to ≤ 5 ng/kg/min Ang II at 30 min is an early predictor of survival benefit [61]. Another analysis reported that a high Ang I/II ratio was associated with more significant norepinephrine requirements and was an independent predictor of mortality [20]. Components of the alternative RAAS have also been associated with outcomes and may serve as biomarkers to inform management. Furthermore, modulation of the alternative RAAS should be investigated to confirm the potential benefit observed in preclinical studies [44].

## Conclusions

The dysregulation of the RAAS in sepsis is a complex and early phenomenon with a likely significant contribution to organ failure and patient outcomes. A better understanding of the pathophysiology and intricacies of the RAAS in septic vasodilatory shock has led to using exogenous angiotensin II as a new drug to treat sepsis. The use of angiotensin II may improve outcomes and organ failure in patients with vasodilatory shock and sepsis. Future research should refine our understanding of the best target population and confirm promising results from initial studies.

## Data Availability

No datasets were generated or analysed during the current study.

## Abbreviations

ACE: Angiotensin converting enzyme
AKI: Acute kidney injury
ANG: Angiotensin
ARB: Angiotensin receptor blocker
ARDS: Acute Respiratory Distress syndrome
DPP3: Dipeptidyl peptidase 3
DVT: Deep vein thrombosis
GFR: Glomerular filtration rate
IFN: Interferon
IL: Interleukin
LPS: Lipopolysaccharide
MAKE: Major adverse kidney events
MAP: Mean arterial pressure
MIP: Macrophage inhibitor protein
MELD: Model for End-Stage Liver Disease
NED: Norepinephrine equivalent dose
NE: Norepinephrine
PE: Pulmonary embolism
RAAS: Renin–angiotensin–aldosterone-system
RBF: Renal blood flow
RRT: Renal replacement therapy
TNF: Tumor necrosis factor
